# Fascia Iliaca Compartment Block for Hip Fractures: Improving Clinical Practice by Audit

**DOI:** 10.7759/cureus.17836

**Published:** 2021-09-08

**Authors:** Isaac C Okereke, Mohamed Abdelmonem

**Affiliations:** 1 Trauma & Orthopaedics, The Royal London Hospital, London, GBR; 2 Trauma & Orthopaedics, University Hospitals, Plymouth NHS Trust, Plymouth, GBR

**Keywords:** fascia iliaca compartment block, clinical audit, hip fracture, ficb, anaesthesia, pain

## Abstract

Background

Hip fractures are the most frequently occurring serious injury in older people. They are the most common reason for people over the age of 65 to need emergency anaesthesia and surgery, and account for the most cause of death following an accident. A fascia iliaca compartment block (FICB) is the injection of anaesthetic agents into the fascia iliaca compartment with the effect of blocking the lumbar plexus via an anterior approach. FICB targets nerves that are in the fascia iliaca compartment that include the femoral nerve and the lateral femoral cutaneous nerve. A FICB is clinically safe and efficient providing consistent analgesic effects irrespective of the performing doctor's experience in managing hip fractures. Clinical audits and feedback are a veritable tool for quality improvement.

Methods

Data from the National Hip Fracture Database (NHFD) for all patients admitted with a hip fracture between October 2018 and May 2019 at a District hospital was interrogated and audited. Results of this audit were discussed in the department's and the Trust's mortality review meetings. In addition, teaching sessions on the safe administration of FICB using the Loss of Resistance (LOR) technique were held for junior doctors. A re-audit was carried out in May 2020 where a retrospective study of patients admitted with a hip fracture over six months from October 2019 to April 2020 was done to assess improvement in compliance rates in the administration of fascia iliaca blocks.

Results

This study showed a statistically significant increase in the number of patients who got a fascia iliaca block on presentation with a fractured neck of the femur from after our second audit (p < 0.00001). There were no complications associated with the administration of FICB to patients with hip fractures.

Conclusions

The administration of FICB for patients with hip fractures by admitting junior doctors using the loss of resistance (LOR) technique is a safe, simple, and rapidly effective pain management method that reduces the need for excessive systemic analgesia and provides consistent simultaneous blockade of the lateral cutaneous femoral and femoral nerves. This study showed that clinical practices could be improved through audits, staff education and by enforcing the proper utilization of clinical proformas to ensure compliance.

## Introduction

Hip fractures are the most frequently occurring serious injury in older people. They are the most common reason for older people to need emergency anaesthesia and surgery and account for the most cause of death following an accident [1]. The incidence rate of hip fractures is about 4.6 per 1,000 adults over 50 and increases exponentially with age in both sexes [2]. With a mortality rate of 10% at one month and between 20% to 35% at one year after injury, fractures of the hip account for more than 1 in 45 of all hospital beds in England and Northern Ireland, and about 1 in 33 in Wales. Only a fraction of patients who have suffered a fracture to their hip return to baseline function, with most becoming dependent and needing long-term care, costing the NHS approximately £1Billion annually (about 1% of the annual NHS budget). In the United States, a patient who has sustained a hip fracture spends up to $40,000 for medical expenses in the first year following the injury, and almost $5,000 in subsequent years, making hip fractures one of the most expensive clinical problems with an estimated 20 billion US dollars spent annually on this injury [3-5]. Standardized mortality ratios show mortality rates to be much higher in people following a hip fracture than in the general population of comparable age and remain raised for months afterwards. The persistently elevated standardized mortality ratio in these patients may be a suggestion of the continuing sequelae of a hip fracture, or, be due to the fact that people who sustain hip fractures might be frailer than the general population [6].

Up to 50%-70% of patients record severe to very-severe pain on pain scoring in the first 24 hours following a hip fracture. The excruciating pain associated with hip fractures and the body's neuroendocrine "stress responses" place significant limitations on patient mobility and negatively impact outcomes. In addition, compromised pulmonary and cardiac function can also occur as a sequential reaction to uncontrolled pain and further exacerbate pre-existing comorbidities [7]. In practice, patients who present with a hip fracture are not operated on emergently due to pressures on the orthopaedic trauma lists in most hospitals. The implication of this is that these patients are moved several times (for imaging, unto the ward beds and trolleys, for pressure area care etc.), further aggravating their pain. The British Orthopaedic Association Standards for Trauma (BOAST) guidelines for the management of fragility fractures recommend "offering immediate and regular analgesia on presentation at hospital and as part of routine nursing observations throughout the admission and ensuring that analgesia is sufficient to allow movements necessary for investigations, nursing care, and rehabilitation" [8].

Current strategies for pain management include systemic analgesics, such as paracetamol, nonsteroidal anti-inflammatory drugs (NSAIDs), narcotics, peripheral nerve blocks, epidurals, and spinal blocks. Although narcotics are used widely in elderly patients who have suffered a hip fracture, they are most effective for static pain, inadequate for controlling dynamic pain [9], and come with a slew of adverse side effects. Adequate analgesia that incorporates a multimodal approach to pain control can reduce the risk of adverse effects and improve postoperative recovery by modifying several of the pathophysiological responses due to pain. Regional blocks like the fascia iliaca compartment blocks (FICB) are now considered an attractive adjunct or alternative for efficacious pain control in this cohort of patients. A Cochrane review in 2002 showed that femoral nerve blocks reduced the degree of pain experienced by patients from a hip fracture and subsequent surgery [10]. In addition, in a randomized controlled study, Beaudoin and colleagues demonstrated that ultrasound-guided FICB as an adjunct to parenteral opioids provides superior pain relief to parenteral opioids alone [11].

In this study, the authors hypothesized that the number of patients receiving FICB for a neck of femur fracture in a UK district hospital could be improved upon by audits, educational/teaching sessions, and employing a clinical proforma in line with the NICE guidelines. (A pre-print of an initial version of this article is online at Research Square, doi:10.21203/rs.3.rs-502228/v1).

## Materials and methods

Data from the National Hip Fracture Database (NHFD) of all patients admitted with a hip fracture between October 2018 and May 2019 was interrogated and audited. In addition, the number of patients receiving FICB, reasons for non-administration of a block, average 24-hour pain scores using the Numeric Rating Scale (NRS), and presentation time in the Emergency department were recorded. Ethical approval was gotten from the Salisbury NHS Clinical audit unit (CA_2020/21/4371).

After the first audit, results were discussed in the department of Trauma & Orthopaedics' and the Trust’s mortality review meetings. A decision for all patients with hip fractures to receive a fascia iliac block on presentation in the emergency department except when contraindicated was taken. Furthermore, the following measures were put in place to improve compliance with the administration of FICB:

The authors implemented small one-to-one teaching sessions on the Loss of Resistance (LOR) technique of FICB administration for Emergency Department and Trauma & Orthopaedics junior doctors.

Proper filling out of the hip fracture clinical proforma was encouraged to ensure FICB were signed off by administering doctors and a reason documented when contra-indicated.

The aim of these interventions was to establish a practice of routine administration of FICB using the LOR technique for patients presenting to the emergency department with a confirmed hip fracture by all junior doctors in order to reduce the over-reliance on opiates for pain control. It was also envisaged that an improvement in rates of early FICB administration would reduce the unwanted and encumbering side effects of opiates overuse and ultimately improve postoperative outcomes for patients. 

Anatomy

The fascia iliaca compartment has the following limits: anteriorly, the posterior surface of the fascia iliaca that covers the iliacus muscle and the whole of the psoas muscle by a medial reflection; the anterior surface of the iliacus and psoas muscles make up the posterior limit; the lower lumbar and sacral vertebrae medially and the inner lip of the iliac crest cranio-laterally; the fascia iliac compartment is continuous with the space between the quadratus lumborum muscle and fascia cranio-medially,

Landmarks and procedure

**Figure 1:**
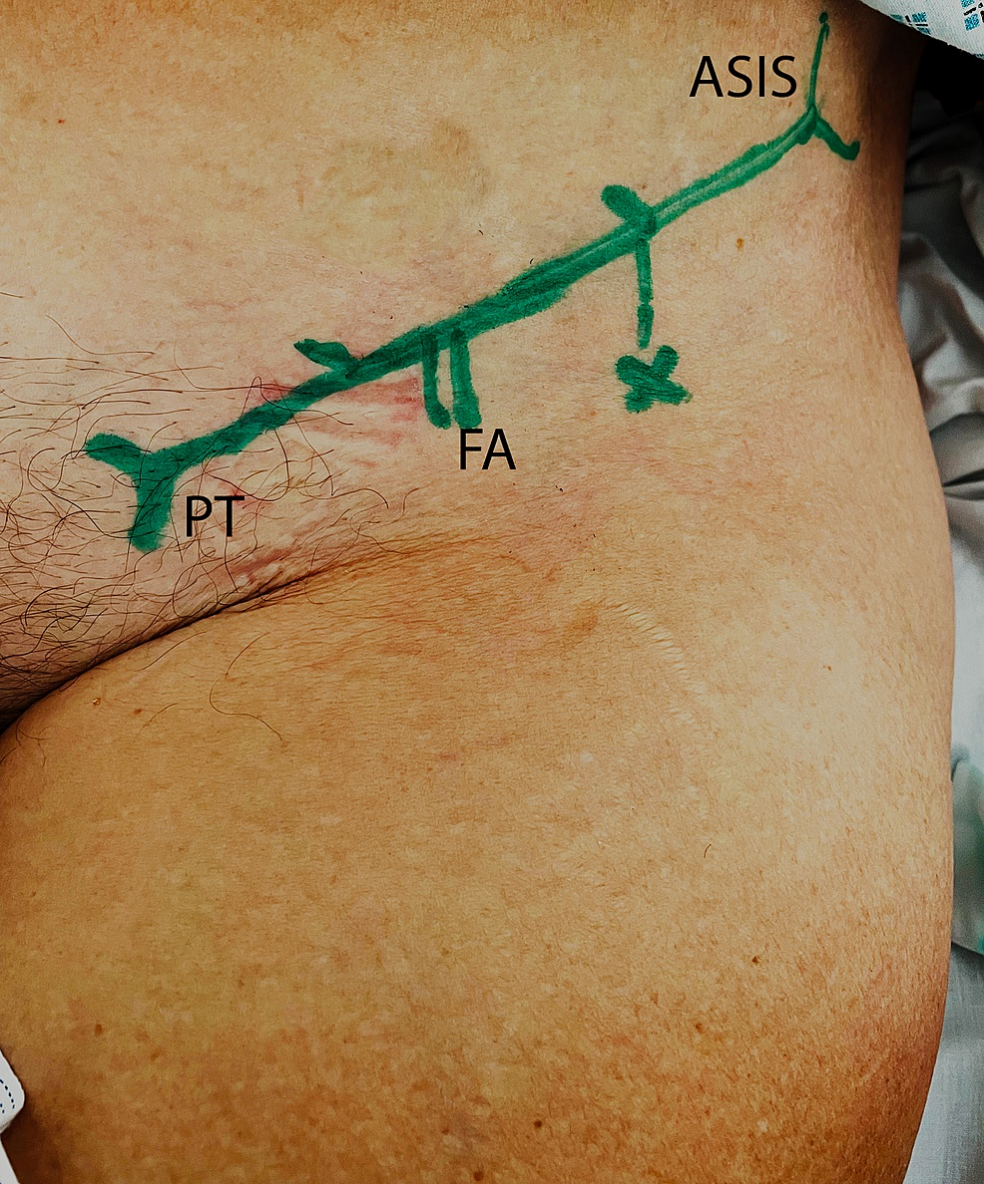
Landmark for FICB injection. ASIS: anterior superior iliac spine; FA: femoral artery; PT: pubic tubercle; FICB: fascia iliaca compartment block.

The landmarks for the procedure are the anterior superior iliac spine (ASIS) and the ipsilateral pubic tubercle (see Figure 1). An imaginary line is drawn between these structures by placing one finger on each of these bony landmarks. This line is divided into thirds, and the puncture site is 2-3 cm caudal to the junction of the medial two-thirds and the lateral third of this line [12]. The FICB is performed under aseptic technique in an appropriately consented patient placed in the supine position to maximize access to the inguinal area. The patient must have an intravenous cannula in-situ and resuscitation equipment nearby. In the LOR technique, a size 18G Tuohy needle is used and a characteristic “2 pops” are felt when penetrating the fasciae lata and iliaca layers indicative of entrance into the fascia iliaca compartment. The syringe is aspirated to rule out any possibility of intravascular infiltration, a weight-dependent volume (20-40 mL: <40 kg 20 ml, 40-80 kg 30 ml, or >80 kg 40 ml) of a long-acting local anaesthetic (0.25% levobupivacaine) is infiltrated into the compartment in 20mls aliquots and with regular monitoring. After injection, the needle is withdrawn and thirty seconds of pressure distal to the injection site is applied to direct the local anaesthetic proximally. The injection site is then dressed. Contraindications to a FICB are: an uncooperative patient, known hypersensitivity reaction to local anaesthesia, a neurological problem affecting the fractured limb, the presence of local infection, previous femoral bypass surgery on ipsilateral limb, and an anti-coagulated patient.

In May 2020, we undertook a retrospective audit of patients admitted with a hip fracture over six months from October 2019 to April 2020 to assess compliance in the administration of fascia iliaca blocks for patients presenting with a hip fracture. The data was again extracted from the NHFD database and scanned notes of patients to determine FICB administration records, reasons for non-administration and average 24-hour pain scores. Also, patient demographics, time of presentation, Abbreviated Mental Test Score (AMTS) scores, and fracture classification into intracapsular were recorded. Dynamic pain scores elicited by gentle logrolling of affected extremity were assessed using the NRS scale between 0 and 10: 0 representing 'no pain at all' and 10 representing 'the worst pain ever possible". Pain scores were recorded pre-FICB administration and then at 30 minutes after a block by the performing Clinician. After this, and over the next 24 hours, pain scoring and monitoring of observations were performed in the orthopaedic wards by healthcare professionals who had had appropriate education and training on how to elicit and record pain scores. A successful block was a pain score at two hours after block administration that was <50% of the pre-FICB score. 

All statistical analysis was performed using SPSS (version 25.0; SPSS Inc, Chicago, IL). The results of each audit were compared and statistically analyzed using the student's t-test to compare continuous variables and the chi-square test for dichotomous variables. A p-value of <0.05 was considered statistically significant.

## Results

**Figure 2:**
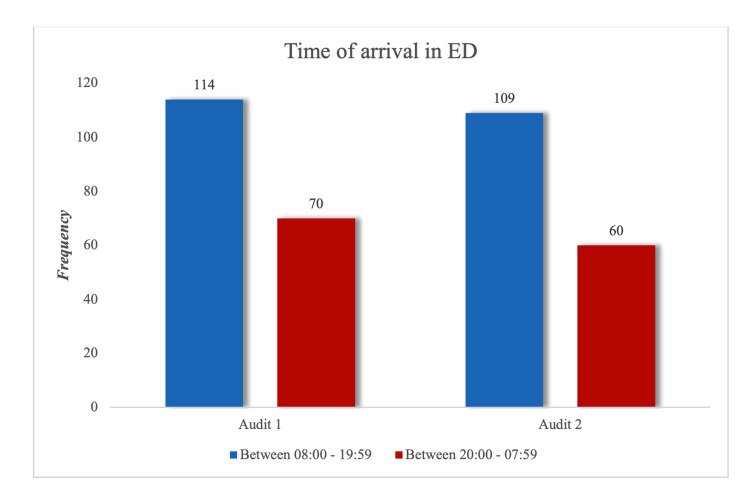
Time of arrival to emergency department.

**Figure 3:**
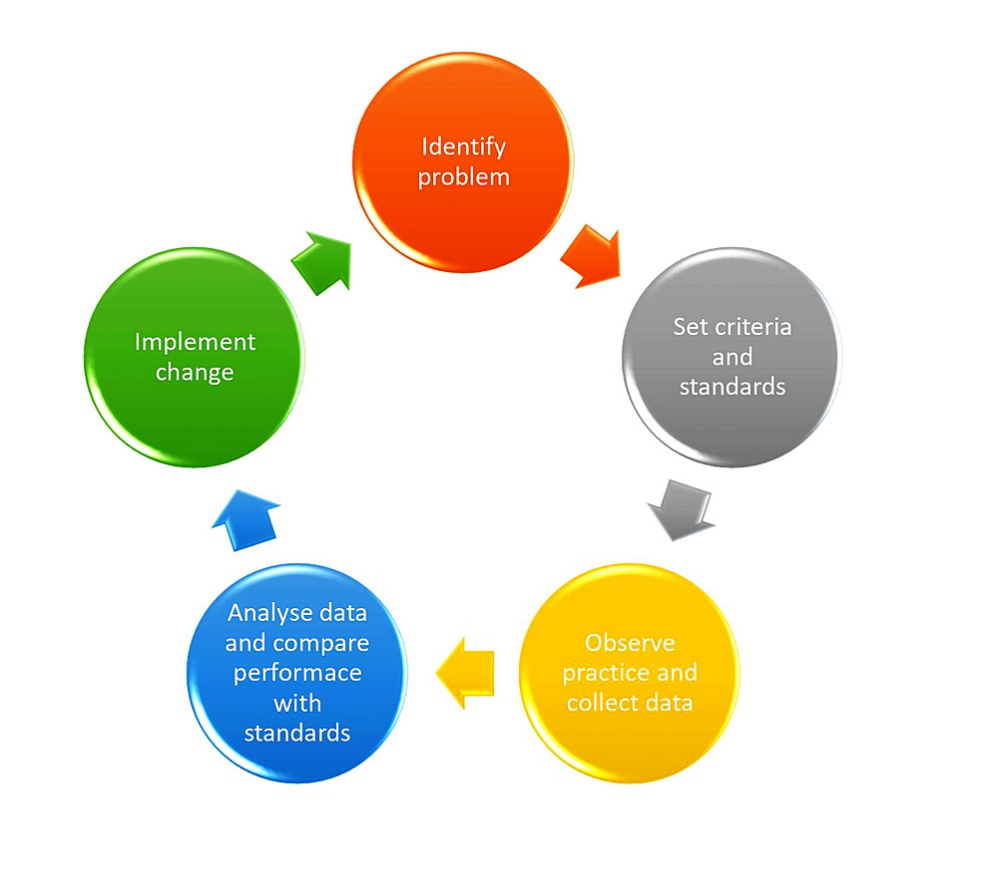
The audit cycle.

The first and second audits had a total of 148 and 169 patients, respectively. Table 1 shows the patient demographics of the two groups. A large proportion of patients in this audit presented to ED between 08:00 and 19:59, as shown in Figure 2 when there are usually more hands in the department and more senior staff on duty than on the night shifts. There were no documented local or systemic complications to fascia iliaca blocks in patients who received blocks in both audits (Figure 3).

**Table 1:** Comparison of demographics and audit outcomes. *Chi-square; ** T-test; FICB, fascia iliaca compartment block; NRS, numeric rating scale.

	First audit (n = 184)	Second audit (n = 169)	p-value
Gender m:f	1:3	1:2.25	0.25412*
Mean age (years)	84.38 ± 7.66	82 ± 7.2	0.0029**
FICB administered n (%)	45 (24.5%)	94 (44.3%)	<0.00001*
Mean pain score pre-FICB (NRS)	8.9	9.5	0.2316**
Mean post-FICB pain score (NRS)	4	3.6	<0.0011**
Reason for non-administration documented	5	3	0.56122*

## Discussion

The assessment and management of pain in elderly patients with a hip fracture can pose a serious challenge due to several reasons that include: increased incidence of complications such as gastrointestinal bleeding and nephrotoxicity from the use of non-steroidal anti-inflammatory drugs (NSAIDs); respiratory depression, sedation, confusion, and constipation from excessive narcotic pain medication usage; difficulty in pain assessment due to frequently co-existing cognitive impairment. 

Effective pain management pre-operatively leads to shorter hospital stays and improved outcomes in elderly patients with a hip fracture. Conversely, patients who experience uncontrolled pain are at a higher risk of developing delirium, have more extended hospital stays and report persistent pain up to six months after a hip fracture [13-15]. The NICE guidelines for managing hip fractures recommend that clinicians consider a multimodal pain control plan, adding nerve blocks if paracetamol and opioids do not provide sufficient preoperative pain relief or limiting opioid dosage [16].

A FICB is the injection of anaesthetic agents into the fascia iliaca compartment with the effect of blocking the lumbar plexus via an anterior approach [17]. The fascia iliaca compartment allows infiltrated local anaesthetic of sufficient volumes to spread to at least two (the femoral and lateral femoral cutaneous nerves) of the three major nerves that supply the medial, anterior, and lateral thigh. Dalens et al. first described FICB in paediatric patients in 1989 [18] when they compared the FICB with the 3-in-1 technique in a study involving 120 children. They reported that while both techniques resulted in similar rates of complete sensory block to the femoral nerve (100%), the FICB provided improved blockade of the lateral femoral cutaneous nerve (92% vs 15%; p < 0.05). Pain from hip fractures is carried by branches from the obturator, sciatic and femoral nerves in accordance with Hilton's law [19]. The FICB may be performed using either a landmark LOR technique or an ultrasound-guided technique. Dalens described the landmark technique as "easy, reliable, requiring no unusual skills or expensive devices, and threatening no vital organ" [20]. In addition, it is a technique that can be taught with minimal instruction and can be performed by junior staff [21]. Stevens and colleagues demonstrated that patients who underwent a FICB used significantly less morphine over 24hours than the control group of patients who used morphine alone. 

In our study, a FICB was administered to patients in the emergency department by the admitting doctor after confirmation of a hip fracture on imaging. All patients had a weight-dependent volume of 0.25% levobupivacaine as anaesthetic agent because of its long-acting duration of effect of about 8-10 hours following a single block [22]. The authors recorded a block success rate of 74%. In the second audit, three patients did not get FICB due to documented contraindications.

Clinical audits are a form of research, the outcomes of which provide new knowledge and a better understanding of the topic being investigated [23]. They are vital aspects of clinical governance and quality improvement. In keeping with the process of audits (Figure 3), the authors identified the poor rate of adequate pain control and heavy use of narcotics in patients presenting with hip fractures as an issue of concern, we observed practice at our local hospital and recorded data. Finally, accumulated data was compared to set standards, the results discussed at mortality review meetings, and change effected and implemented. With less than 75% of hip fracture patients receiving their operation within 36 hours as recommended in the NICE guidelines, the safe administration of FICB should form part of the admission protocol for hip fractures. 

There are several limitations to this study. Both audits were conducted in a retrospective manner. We did not directly compare FICB administration to other pain control modalities; the reason for this being our study focused on the use of clinical audits to effect change in clinical practice. This also meant that not a lot of emphasis was placed on the effectiveness or not of FICB as this is already well documented in previous studies [24-26]. Additionally, our data did not include the number and grades of junior doctors who received teaching on FICB administration. This is important as most junior doctors spend about 6months in each clinical posting. The duration between our first and second audits implies that a different set of junior doctors would have been working in ED and orthopaedics departments.

## Conclusions

The administration of fascia iliaca blocks for hip fractures is a safe, simple, and rapidly effective pain management method that reduces the need for systemic analgesia. Furthermore, FICB provides consistent simultaneous blockade of the lateral cutaneous femoral and femoral nerves irrespective of the performing doctor's experience of managing hip fractures. This study showed the effectiveness of auditing clinical practice against standards, giving feedback, and modifying practice amongst healthcare professionals to improve patient care.
